# Evaluation of the hypothalamo-pituitary-adrenal axis during the post-COVID-19 period in patients treated with steroids during the illness

**DOI:** 10.20945/2359-4292-2022-0207

**Published:** 2023-11-10

**Authors:** Shyam Sundar C M, Jayanthy Ramesh

**Affiliations:** 1 NTR University of Health Sciences Andhra Medical College Department of Endocrinology Visakhapatnam India NTR University of Health Sciences, Department of Endocrinology, Andhra Medical College, Visakhapatnam, India

**Keywords:** Hypothalamo-pituitary-adrenal axis, post-COVID, COVID-19, steroid treatment

## Abstract

**Objective:**

COVID-19 is a multisystem immunoinflammatory disorder, and the hypothalamo–pituitary-adrenal (HPA) axis may be affected by SARS-CoV-2 as well as by steroid treatment during the illness. Information on the HPA axis after recovering from COVID-19, especially in those treated with steroids, is sparse. Hence, this study was performed to evaluate the hypothalamo-pituitary-adrenal axis during the post-COVID-19 period in patients treated with steroids during the illness.

**Subject and methods:**

This prospective study involved 60 patients aged 18-60 years who had recovered from moderate or severe COVID-19 and had received steroid treatment during the illness. The HPA axis was assessed with a low-dose (1 mcg) adrenocorticotropic hormone stimulation test at 3, 6 and 9 months in the post-COVID period.

**Results:**

The HPA axis was suppressed in 31.66% of the patients at 3 months and 5% at 6 months; however, all patients recovered at 9 months. Cumulative steroid use during the illness was inversely correlated with stimulated cortisol at 3 months in the post-COVID period. Fatigue was present in 58.33% of the patients at 3 months and was more prevalent in those with HPA axis suppression.

**Conclusion:**

Nearly one-third of the patients with moderate to severe COVID-19 who were treated with steroids had suppressed HPA axis at 3 months, with gradual recovery over a period of 9 months. Cumulative steroid equivalent dose, but not disease severity, was predictive of HPA axis suppression at 3 months.

## INTRODUCTION

Severe acute respiratory syndrome coronavirus 2 (SARS-CoV-2) causes coronavirus disease 2019 (COVID-19) in humans. Approximately 15% of the infected patients develop moderate to severe pulmonary disease as a result of the multisystem immunoinflammatory response caused by COVID-19 (1). The virus enters the target cells in multiple organs, including the pituitary and adrenal glands, via the ACE2 receptor. Direct injury to the adrenal and pituitary cells by the virus can result in primary or secondary adrenal insufficiency (2–4).

Research has shown that glucocorticoid treatment exerts beneficial effects on the immunoinflammatory reaction in moderate and severe COVID-19 by reducing the mortality rate and the duration of hospital stay (5). This finding resulted in widespread, albeit indiscriminate, glucocorticoid use during the COVID-19 pandemic. The effect of steroid use on the hypothalamo–pituitary-adrenal (HPA) axis during recovery from COVID-19 warrants attention as both adrenal insufficiencies and long COVID have similar symptomatology (6). Information on the HPA axis after recovering from COVID-19, especially in those treated with steroids, is sparse. Hence, this study attempted to evaluate the HPA axis among patients who recovered from moderate to severe COVID-19 and were treated with steroids.

## PATIENTS AND METHODS

This study was conducted in the Department of Endocrinology, King George Hospital, Andhra Pradesh, India, between May and December 2021. After obtaining institutional ethics committee clearance, the trial was registered with the Clinical Trial Registry of India with the following ITCRP ID: ITRCP-CTRI/2021/05/033799. Inpatient clinical and laboratory data of those admitted between 1 May 2021 and 15 June 2021 were retrieved from the medical records for the study. Reverse transcription-polymerase chain reaction-positive hospitalized patients with COVID-19 aged 18-60 years with clinical features suggestive of moderate or severe disease and diagnosed as per the updated detailed clinical management protocol for adult patients with COVID-19 issued by the Ministry of Health and Family Welfare, Government of India, dated 14-01-2021 and 24-5-2021 (7,8) and only those who received steroid treatment for less than 14 days and recovered from the illness were included in the study.

The criteria used to define moderate and severe COVID-19 were as follows: moderate pneumonia with no signs of severe disease: adults with the presence of clinical features of dyspnoea and/or hypoxia, fever, and cough, including SpO_2_ ≤ 93% in room air, respiratory rate ≥ 24 breaths per minute; adults with severe pneumonia with clinical signs of pneumonia plus one of the following: respiratory rate > 30 breaths/min, severe respiratory distress, SpO_2_ < 90% in room air; acute respiratory distress syndrome (ARDS) (onset: new or worsening respiratory symptoms within 1 week of known clinical insult; chest imaging (chest X-ray and portable bedside lung ultrasound): bilateral opacities not fully explained by effusions, lobar or lung collapse or nodules; origin of pulmonary infiltrates or respiratory failure not fully explained by cardiac failure or fluid overload; need objective assessment (e.g. echocardiography) to exclude hydrostatic cause of infiltrates/oedema if no risk factor is present; oxygenation impairment in adults: mild ARDS: 200 mmHg < PaO_2_/FiO_2_ ≤ 300 mmHg (with PEEP or CPAP ≥ 5 cm H_2_O) moderate ARDS: 100 mmHg < PaO_2_/FiO_2_ ≤ 200 mmHg with PEEP ≥5 cm H_2_O) severe ARDS: PaO_2_/FiO_2_ ≤ 100 mmHg with PEEP ≥ 5 cm H_2_O), sepsis resulting in acute life-threatening multi-organ dysfunction; septic shock (persisting hypotension despite volume resuscitation, requiring vasopressors to maintain MAP ≥ 65 mmHg and serum lactate level > 2 mmol/L (8).

Pregnant women, those receiving glucocorticoids for any indication before hospital admission, those with primary/secondary adrenal insufficiencies, cortisol excess states, chronic kidney disease, chronic liver disease, coronary artery disease, cerebrovascular accident, anti-epileptic therapy or COPD and those with a history of smoking or chronic alcoholism were also excluded from the study.

The patients were telephonically contacted and informed about the nature, objective and procedure of the study and were invited to participate in it. Those who provided written informed consent for participation in the study were included. Information was collected via prepared proforma for each patient. Data regarding clinical characteristics, biochemical parameters, duration and type of steroid treatment, dose and cumulative dose of steroid treatment received during the hospital stay were noted from the case records. The overall duration of steroid exposure or the cumulative steroid equivalent dose was analysed for each patient. The cumulative steroid equivalent dose in mg was calculated using the formula cumulative dose of dexamethasone in mg × 5.3, and the total dexamethasone dose was converted to methylprednisolone (9,10).

At the first visit, i.e. 3 months into the post-COVID-19 period, clinical details regarding weakness, lethargy, body pain, postural giddiness, breathlessness and cough were documented. Demographic parameters and vital data, including height, waist circumference, pulse rate and blood pressure in the supine and standing positions at 3 minutes were recorded. Fasting venous blood sample was collected in ethylenediaminetetraacetic acid and serum vacutainer via venipuncture between 8:00 and 9:00 am. Hemogram, plasma glucose, serum creatinine, blood urea, serum electrolytes, SGOT and SGPT were analysed on the same day. The serum was separated by centrifugation at 2,000 rpm for 5 minutes and stored at −80 °C for analysis of basal cortisol. One microgram (mcg) of Synacthen (cosyntropin) was administered intravenously as part of a low-dose ACTH stimulation test, and a sample for serum cortisol estimation was obtained 30 minutes after the injection (referred to as stimulated cortisol hereafter). Cosyntropin 1 mcg was prepared (0.4 mL contained 1 mcg) by diluting 250 mcg of cosyntropin (1 mL) in 99 mL of normal saline, stored at 2-8 °C and used within 4 weeks after reconstitution (11).

Serum cortisol was measured using the Access cortisol assay, which is a competitive binding immunoenzymatic assay run on the Beckman Coulter UniCel DxI 800. The intra-assay variability was 3.4%-4.7% and inter-assay variability was 4.1%-5.7% for concentrations ranging from 4.4 μg/dL to 35.3 μg/dL. The lowest detectable level of cortisol distinguishable from zero (Access Cortisol Calibrator S0) with 95% confidence is 0.4 µg/dL (11 nmol/L) (12).

Basal 8:00 am serum cortisol < 5 µg/dL was considered hypocortisolism owing to suppressed HPA axis, and values > 15 mcg/dL were considered normal. Normal stimulated cortisol response after low-dose ACTH stimulation was defined as serum cortisol ≥ 18 mcg/dL and suppressed HPA axis of < 18 µg/dL (13).

In patients with stimulated cortisol of < 18 mcg at 3 months, low-dose ACTH stimulation test was repeated after another 3 months, i.e. 6 months after recovery from COVID-19.

### Statistical analysis

Data were entered in a Microsoft Excel spreadsheet, version 2013, and analysed using SPSS software, version 19.0 (International Business Machines Corporation, 2010). Categorical variables were described using frequencies and percentages. Data were expressed as mean and standard deviation or median and interquartile range (IQR), as appropriate. Means and standard deviations were compared using the student's t-test, whereas medians and IQR were compared using the non-parametric Mann-Whitney U test when comparing two groups and the Kruskal–Wallis test when comparing more than two groups. Proportions were compared using the χ2 test. Association between non-normally distributed variables was derived using Spearman's correlation coefficient. A p-value of <0.05 was considered significant.

## RESULTS

### Baseline parameters

A total of 101 eligible patients were telephonically invited to participate in the study, and 60 of them who were willing to participate were included. At the time of hospital admission, moderate COVID-19 was present in 34 patients (21 men and 13 women) and severe illness in 26 patients (16 men and 10 women). The mean age of the study population was 41.8 ± 8.72 years (range: 23-56 years). Before inpatient admission, the mean duration of symptoms was 6.2 ± 2.83 days. The mean SpO_2_ at admission was 88.71 ± 6.55, and the mean duration of hospital stay was 8.53 ± 3.47 days. Diabetes and hypertension were present in 13 and 5 patients respectively, and 6 patients had both. The mean total leukocyte count was 9475 ± 3799 cells/µL (normal range: 4,500-11,000 cells/µL (4.5-11.0). The other parameters were as follows: lactate dehydrogenase (LDH) 702 ± 328 IU/L (normal range: 105-333), D dimer 0.53 ± 0.39 mcg/mL (normal: <0.50 mcg/mL), ferritin 342.05 ± 301 ng/mL (normal range: 12-300 ng/mL for men and 12-150 ng/mL for women), and C-reactive protein (CRP) 7.05 ± 11.03 mg/L (normal: <10 mg/L). During the inpatient treatment, 60% of the patients received dexamethasone and 40% received methylprednisolone. The average duration of steroid use was 6.3 ± 3.2 days, and the calculated cumulative steroid equivalent dose was 397.28 ± 321.3 mg of methylprednisolone. All patients received standard-of-care treatment during hospitalization.

### Assessment at 3 months post-COVID-19 (i.e. initial visit of the study)

The mean body mass index (BMI) was 25.91 ± 3.53 kg/m^2^. It was observed that 58.37% of the patients had fatigue, followed by mild dyspnoea on exertion (NYHA1) in 30% and myalgia in 10%. However, none of them had postural giddiness, nausea, vomiting or abdominal pain. The mean pulse rate, systolic blood pressure and diastolic blood pressure were 79.7 ± 5.7 beats per minute, 126 ± 5 mmHg and 78 ± 6 mmHg, respectively, and none of them had postural fall in blood pressure. Dysglycaemia was seen in four individuals who were previously not known to have diabetes. The mean total leukocyte count (TLC) was 7,337 ± 2,489 cell/mm^3^, neutrophil count was 56% ± 7%, lymphocyte count was 38% ± 7% and platelet count was 2.5 ± 0.6 lakhs/mm^3^. The mean 8:00 am basal cortisol was 10.42 ± 2.71 mcg/dL; 55 patients had values of 5-15 mcg/dL, and only 5 patients had values of > 15 mcg/dL. None of the patients had values of < 5 mcg/dL. In the low-dose (1 mcg) ACTH stimulation test, 19 of the 60 patients (31.66%) had stimulated cortisol of < 18 mcg/dL and 41 patients (68.33%) had ≥ 18 mcg/dl. The study cohort was categorized into two groups based on the stimulated cortisol values, as follows: Group 1: sub-normal response or suppressed axis, i.e. < 18 mcg/dL (19 patients) and Group 2: normal or recovered HPA axis, i.e. ≥ 18 mcg/dL (41 patients) at 3 months. No statistically significant difference was noted between the two groups in terms of sex distribution, mean age, SpO_2_ at admission, severity of the illness or duration of hospital stay.

The TLC at admission was lower (9,865 ± 4,049 cells/mm^3^) in those with suppressed HPA axis than in those with normal axis (8,631 ± 2,854 cells/mm^3^); the difference, however, was not statistically significant (p = 0.181). At 3 months post-COVID-19, no significant linear correlation was noted between TLC at admission and stimulated cortisol level (p = 0.738). Furthermore, the markers of COVID-19 severity, such as CRP, LDH, D dimer and ferritin were not significantly different between the two groups (Table 1). Diabetes at admission was reported in 47% (9 of the 19 patients) in the suppressed HPA axis group and 25% (10 of the 41 patients) in the normal HPA axis group; nevertheless, the difference was not statistically significant (p = 0.071). Hypertension was also not a significant contributing factor. Similarly, no significant difference was seen when the mean BMI, waist circumference and TLC were compared between those with suppressed and normal HPA axis (Table 2).

**Table 1:** Clinical and Biochemical characteristics at hospital admission among those with Suppressed and Normal HPA axes (retrospective analysis)

Parameter		Suppressed HPA axis (n = 19)	Normal HPA axis (n = 41)	P-value
Male: Female		1.37:1	1.73:1	0.68
Age (years)		40.94 ± 9.51	42.21 ± 8.42	0.18
BMI (kg/m^2^)		27.12 ± 4.6	25.46 ± 2.81	0.04
Diabetes mellitus		9 (47.3%)	10 (24.3%)	0.07
Hypertension		4 (21%)	5 (12.2%)	0.48
Severity of illness				0.68
	Moderate	10	24	NA
	Severe	9	17	NA
Hospital stay (days)		9.1 ± 2.92	8.2 ± 3.69	0.58
spO2 (%)		89.21 ± 4.81	81.88 ± 7.2	0.29
TLC (cells per microliter)		8,631 ± 2,854	9,865 ± 4,049	0.18
Platelets (lakhs per microliter)		2.51 ± 0.96	2.30 ± 1.03	0.84
LDH (IU/L)		666.8 ± 379.1	720.8 ± 303.5	0.60
D Dimer (ug/mL)		0.57 ± 0.49	0.52 ± 0.34	0.27
Ferritin (ng/mL)		325.1 ± 266.5	349.7 ± 318.8	0.53

**Table 2:** HPA axis and clinical and biochemical characteristics at first visit i.e. at 3-month post covid and steroid usage data

Parameters	Suppressed HPA axis (n = 19)	Recovered HPA axis (n = 41)	P-value
General weakness	13 (68.5%)	22 (53.6%)	0.21
Waist Circumference (cm)	90.15 ± 12.62	41 ± 6.37	0.47
TLC (per microliter)	8,105 ± 3,090	6,768 ± 2,210	0.05
Basal cortisol µg/dL	9.09 ± 2.1	11.04 ± 2.76	0.004
Stimulated cortisol µg/dL	13.97 ± 2.2	22.47 ± 3.77	0.001
Dexamethasone (no.)	8 (42.1%)	28 (68.3%)	0.05
Methylprednisolone (no.)	11 (57.9%)	13 (31.7%)	0.05
Duration of steroid use (days)	6.89 ± 2.86	6.02 ± 1.99	0.17
Cumulative equivalent steroid dose (mg)	554.6 ± 473.6	324.4 ± 185.7	0.002

**Figure 1:**
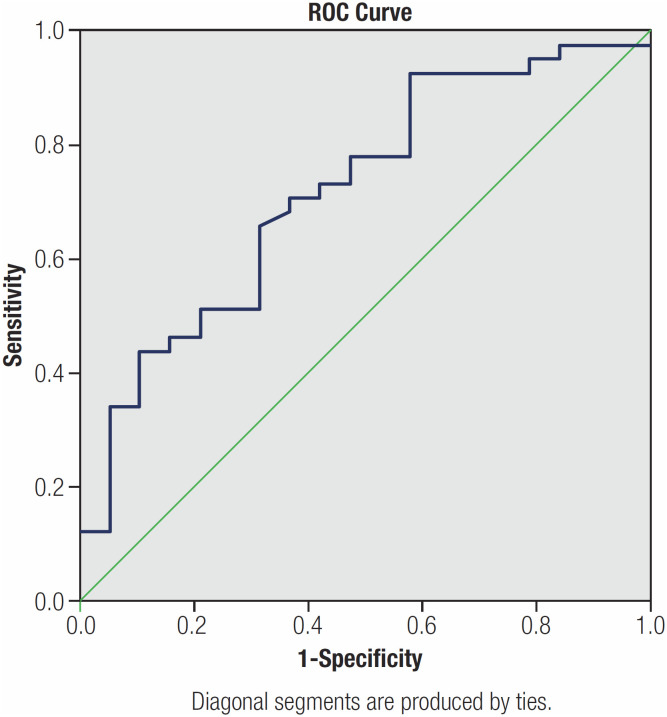
ROC, at an AUC cut-off of 0.718. Basal cortisol 9.79 mcg/dL predicted a subnormal peak stimulatory response.

The mean basal 8:00 am cortisol was significantly lower (p = 0.004) in those with suppressed HPA axis than in those with normal axis, and the mean stimulated cortisol was also significantly lower in those with suppressed axis (p < 0.001) than in those with a normal axis. Basal cortisol levels of < 5.79 µg/dL and > 12.2 µg/dL had 98% and 95% sensitivity, respectively, in predicting the suppressed and normal cortisol responses to 1-ug ACTH stimulation but had very low specificity. However, in receiver operating characteristic curve analysis, at an area under the curve cut-off of 0.718, a basal cortisol value of 9.79 µg/dL predicted a subnormal peak stimulatory response to low-dose ACTH, with a maximum sensitivity of 69% and specificity of 64% (Figure 1).

**Figure 2:**
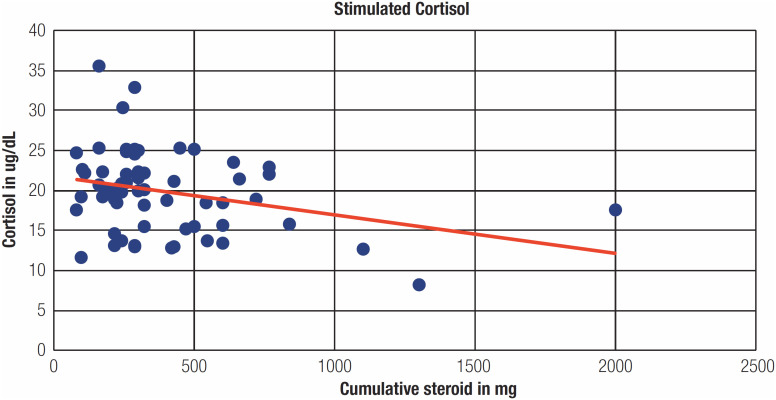
Inverse linear relation between cumulative steroid dosage and Stimulated cortisol at 3 months (correlation coefficient −0.295).

When the steroid usage data were analysed, 57.9% and 31.7% of the patients received methylprednisolone among those with suppressed and normal HPA axis, respectively. The difference was statistically significant (p = 0.05). The mean cumulative equivalent steroid dose usage during admission was significantly higher in those with HPA axis suppression than in those with normal axis (554.6 *vs.* 324.4 mg) (p = 0.009) (Table 2). Moreover, a modestly significant inverse correlation was observed between the cumulative steroid dose and the values of stimulated cortisol at 3 months (p = 0.022; correlation coefficient: −0.295) (Figure 2). However, there was no statistically significant difference between the duration of steroid use and the HPA axis at 3 months.

The mean 8:00 am cortisol was significantly lower in those who reported fatigue 3 months into the post-COVID-19 period than in those without fatigue (p = 0.05). Fatigue was more prevalent (68.5%) among those with suppressed HPA axis than in those with a normal axis (41.4%), but the difference was not statistically significant (p = 0.214). The prevalence of fatigue was higher in women than in men (p = 0.014). Age, SpO_2_ at admission, TLC, serum ferritin, CRP and stimulated cortisol did not have any relationship with the presence of fatigue at 3 months (Table 3).

**Table 3:** Post Covid Fatigue analysis: Clinical and biochemical characteristics

Parameters	Fatigue present n= 35	No fatigue n= 25	P-value
Age	41.45	42.36	0.68
Gender (M: F)	17:18	20:5	0.014
Severity (mod:sev)	20:15	14:11	0.93
BMI	26.43 ± 3.88	25.3 ± 2.9	0.20
Haemoglobin (g/dL)	12.16 ± 1.6	12.8 ±1.36	0.11
TLC (cell/mm^3^)	9142 ± 2502	9600 ± 2709	0.42
D dimer	0.52 ± 0.39	0.55 ± 0.38	0.76
LDH	776.57 ± 27.9	569.6 ± 52.32	0.04
Steroid used methylprednisolone: dexamethasone	13:22	11:14	0.59
Steroid equivalent dose (mg)	452.17 ± 384	320.42 ± 186	0.12
8am cortisol (mcg/dL)	9.83 ± 2.68	11.24 ± 2.58	0.05
Stimulated cortisol (mcg/dL)	18.97 ± 5.26	20.91 ± 4.97	0.15

### Assessment at 6 months and 9 months into the post-COVID-19 period

All 19 patients with suppressed HPA axis at 3 months were followed up till 6 months into the post-COVID-19 period. However, none of them had hypotension or postural fall in blood pressure. The mean basal cortisol was 9.27 ± 2.67 µg/dL and stimulated cortisol was 20.59 ± 3.43 µg/dL. Only 3 of the 19 patients (15.7%) had persistent HPA axis suppression at 6 months without any symptoms of hypocortisolism. During supervised follow-up for another 3 months, all of them showed complete recovery of the HPA axis at 9 months post-COVID-19.

## DISCUSSION

The morbidity and mortality associated with severe COVID-19 could be attributed to its progressive multisystemic involvement and immunoinflammatory vascular injury. Isolated case reports have been published on new-onset adrenal insufficiency during or after severe COVID-19; however, steroid usage during the illness has not been discussed (14–17). In addition to the standard care of treatment, the use of steroids has been linked to improved recovery and reduced mortality, especially in patients with moderate to severe COVID-19 (5). Data on the long-term effects of steroid treatment on the HPA axis suppression/recovery status in these patients is sparse. Overt or subclinical adrenal insufficiency may be a consequence of HPA axis suppression. Long COVID-19 or post-COVID-19 syndrome is characterized by fatigue, weakness and dizziness and may be associated with subclinical adrenal insufficiency (18,19). Hence, we planned to explore the contributory role of HPA axis suppression in these patients.

Clarke and cols. evaluated adrenal function using a 250 mcg short Synacthen test (SST) at 3 months in 70 individuals who recovered from COVID-19. The authors documented normal adrenal status in all subjects irrespective of the severity of illness or steroid usage. No association was observed between the presence of persistent fatigue and adrenal function in the post-COVID-19 period (20). In contrast, we used a low-dose 1 mcg ACTH stimulation test to evaluate the status of the HPA axis at 3, 6 and 9 months after recovery from COVID-19. The results indicated that 31.66% of the patients had HPA axis suppression in the form of subclinical adrenal insufficiency at 3 months. All patients in our study had received steroids, either dexamethasone or methylprednisolone, in varying doses and duration. Correlation studies on the type of steroid, duration of steroid usage, mean cumulative steroid dosage and HPA axis suppression were performed at 3 months in our study.

An earlier study on the survivors of the severe acute respiratory syndrome (SARS) outbreak in 2003 by Leow and cols. showed the presence of hypocortisolism at 3 months in 39.3% of the patients tested with 1 mcg SST, and only 10 of the 61 patients had received steroid treatment (21). However, ours is the first prospective study to evaluate the HPA axis using 1 mcg low-dose SST in the post-COVID-19 period in the setting of the pandemic. The use of supra-physiologic doses of ACTH (250 mcg SST), as done by Clarke and cols., can stimulate the adrenal glands in those with recent-onset HPA dysfunction, thus leading to false-negative results (20). The use of a low-dose Synacthen stimulation test has been shown to have higher sensitivity for the diagnosis of HPA axis suppression (4,22,23). The cortisol values obtained with low-dose SST were closely correlated with those obtained from the gold standard insulin tolerance test (24). A meta-analysis of studies on SST showed that low-dose (1 mcg) SST has better sensitivity for secondary hypocortisolism and that standard-dose SST has better specificity (25–28).

The mean cumulative steroid equivalent dose was significantly higher in those with severe illness, which suggests the contribution of high doses to the HPA axis suppression (Table 3). To determine whether COVID-19 or the steroid usage resulted in hypocortisolism, a comparative control group without steroid use would have been ideal. However, as per our institutional treatment protocol during the pandemic, all hospitalized patients with moderate or severe illness were treated with steroids.

Dexamethasone has been reported to have higher suppressive effect on the HPA axis compared with methylprednisolone (29). However, in our study, a high prevalence of hypocortisolism at 3 months was reported in those treated with methylprednisolone than in those treated with dexamethasone (Table 3). As our study was not powered to determine the difference in outcome between different steroids, the above finding needs to be confirmed with further studies. The higher mean cumulative steroid dose in patients treated with methylprednisolone in our study could explain the higher prevalence of HPA axis suppression in them. The time taken for HPA axis recovery following the administration of different doses of steroid and different duration of treatment has shown that the recovery period ranges from 14 days to 1 year (30,31). A study on the HPA axis recovery after short-term methylprednisolone usage in nine patients with chronic obstructive pulmonary disease by Schuetz and cols. revealed that 33% of the patients had suppressed HPA axis 3 weeks after the last steroid dose, as tested with 1 mcg SST, although long-term follow-up data were unavailable (32). Our study performed in the post-COVID-19 setting demonstrated that short-term steroid therapy with a cumulative dose of 397 mg resulted in hypocortisolism in 31.6% of the patients at 3 months, with progressive and complete recovery over a period of 6-9 months.

Henzen and cols. reported that 45.3% (34 of 75) of the patients who received an equivalent cumulative dose of 150-5,027 mg prednisolone over 5-30 days had HPA axis dysfunction 2 days after steroid therapy cessation. Most patients recovered within 14 days, and in two patients, the HPA axis remained suppressed at 6 months (33). However, in our study, all patients received steroids for less than 14 days and 31.66% had HPA axis suppression at 3 months, with a majority (84.5%) recovering at 6 months and all of them at 9 months. Age, sex, severity of the illness, presence of comorbidities and type and dose of steroid usage did not have any predictivity on the HPA axis recovery at 6 months.

Basal cortisol as a marker of HPA axis recovery has been investigated in earlier studies. In our study, although basal cortisol levels of < 5.79 µg/dL and > 12.2 µg/dL had 98% and 95% sensitivity, respectively, they exhibited low specificity in predicting suppressed and normal cortisol responses to 1 µg low-dose ACTH stimulation. These values are close to those obtained by Schuetz and cols. (5.43 µg/dL and 14.49 µg/dL, respectively) and congruent to the guidelines of adrenal insufficiency (32,13). Although our sample size was small, a single basal cortisol value of 9.79 µg/dL had a sensitivity of 69% and specificity of 67% in predicting subnormal peak response after low-dose Synacthen stimulation (Figure 1).

Single morning cortisol values are not recommended to rule out adrenal insufficiency although studies have reported 100% sensitivity for values in the range of 10.3-17 µg/dL to rule out adrenal insufficiency (26,27). In general, morning cortisol of < 5 µg/dL suggests adrenal insufficiency in those individuals who are not on exogenous steroids. However, with current laboratory assays, values of 4.1-4.7 µg/dL have also been observed in normal individuals. A low-dose SST is recommended to rule out adrenal insufficiency in resource-poor settings; alternatively, paired 8:00 am cortisol of < 5 µg/dL and ACTH values of less than twice the upper limit can be used to diagnose HPA axis suppression (13). In our study, age, sex, severity of the illness, presence of comorbidities, immunohaematological parameters and markers of inflammation did not have any influence on the HPA axis status at 3 months (Table 1).

Recent literature on long-COVID-19 syndrome suggests that fatigue is a common symptom, and HPA axis dysfunction could be a causative factor (34). Townsend and cols. (35) reported fatigue in 54% of the 128 participants at 3 months into the post-COVID-19 period. The severity of the illness, markers of inflammation and markers of cell turnover did not influence the prevalence of fatigue at 3 months, but the HPA axis was not evaluated in the study (29). In contrast, our findings indicated that female sex and higher serum LDH at admission were associated with the persistence of fatigue 3 months after recovery from the illness. The mean 8:00 am cortisol was significantly low in those with fatigue compared with those who were not fatigued. Of those with HPA axis suppression, 68.5% reported fatigue compared with 41.4% in those with normal HPA axis, but the difference was not statistically significant. At 9 months post-COVID-19, none of the subjects reported fatigue and the HPA axis was normal. Leow and cols., in their study on patients who recovered from SARS, documented a similar relationship between fatigue and low cortisol levels (21). Using 1 mcg low-dose SST, the researchers identified that 87% of those with suppressed HPA axis experienced fatigue. Another study reported a high prevalence of fatigue (73%) 3 months after recovery from COVID-19, and none of the patients had HPA axis suppression in the 250 mcg SST (20). Although post-COVID-19 fatigue was reported frequently among those with HPA axis suppression in our study, no statistical significance was noted (p = 0.15).

In summary, in this prospective study conducted during the post-COVID-19 period in patients with moderate and severe illness who were treated with glucocorticoids, HPA axis suppression was observed in 31.66% and 5% of the total cohort at 3 and 6 months, respectively, and all the patients recovered at 9 months. Cumulative steroid doses at admission were predictive of suppressed HPA axis at 3 months. All patients who showed a subnormal response with 1 µg SST were clinically eucortisolic with no hypotension, and some of them had non-specific symptoms of fatigue and weakness. Hence, they were followed up with close supervision without any steroid treatment, and the HPA axis recovered completely in all the patients.

### Limitations

Our study has certain limitations. First of all, ours was a single-centre descriptive pilot study; large multi-centre studies are needed to obtain a better understanding. Moreover, admission cortisol values before commencing steroid treatment were not available. The absence of a control patient population who were not treated with steroids is another limitation. As patients admitted to the hospital constituted our study cohort, all of them had received steroids during the hospital stay. In addition, fatigue was evaluated merely by its presence or absence, and a fatigue score was not used.

### Strengths

Ours is the first prospective observational study to evaluate the HPA axis in the post-COVID-19 period using the low-dose (1 mcg) ACTH stimulation test. The findings from our research have reinforced the concept that adrenal dysfunction might contribute to the long-COVID-19 syndrome. We investigated the effects of clinical, biochemical and immunoinflammatory parameters at admission and the treatment administered on the HPA axis at 3 months. The extended follow-up period of up to 9 months aided in understanding the HPA axis recovery pattern.

In conclusion, nearly one-third of individuals with moderate to severe COVID-19 who were treated with steroids had suppressed HPA axis at 3 months and 5% at 6 months into the post-COVID-19 period, but all of them recovered at 9 months. Cumulative steroid equivalent dose, but not disease severity, was predictive of HPA axis suppression at 3 months.
